# Application of Sucrose Acetate Isobutyrate in Development of Co-Amorphous Formulations of Tacrolimus for Bioavailability Enhancement

**DOI:** 10.3390/pharmaceutics15051442

**Published:** 2023-05-09

**Authors:** Eman M. Mohamed, Sathish Dharani, Mohammad T. H. Nutan, Phillip Cook, Rajendran Arunagiri, Mansoor A. Khan, Ziyaur Rahman

**Affiliations:** 1 Irma Lerma Rangel School of Pharmacy, Texas A&M Health Science Center, Texas A&M University, College Station, TX 77843, USA; eman_nabil24@tamu.edu (E.M.M.);; 2 Department of Pharmaceutics, Faculty of Pharmacy, Beni-Suef University, Beni-Suef 62514, Egypt; 3 Irma Lerma Rangel School of Pharmacy, Texas A&M Health Science Center, Texas A&M University, Kingsville, TX 78363, USA; 4 Eastman Chemical Company, Kingsport, TN 37662, USA

**Keywords:** tacrolimus, sucrose acetate isobutyrate, amorphous solid dispersion, dissolution, stability, pharmacokinetics

## Abstract

The focus of the present work was to develop co-amorphous dispersion (CAD) formulations of tacrolimus (TAC) using sucrose acetate isobutyrate as a carrier, evaluate by in vitro and in vivo methods and compare its performance with hydroxypropyl methylcellulose (HPMC) based amorphous solid dispersion (ASD) formulation. CAD and ASD formulations were prepared by solvent evaporation method followed by characterization by Fourier transformed infrared spectroscopy, X-ray powder diffraction (XRPD), differential scanning calorimetry (DSC), dissolution, stability, and pharmacokinetics. XRPD and DSC indicated amorphous phase transformation of the drug in the CAD and ASD formulations, and dissolved more than 85% of the drug in 90 min. No drug crystallization was observed in the thermogram and diffractogram of the formulations after storage at 25 °C/60% RH and 40 °C/75% RH. No significant change in the dissolution profile was observed after and before storage. SAIB-based CAD and HPMC-based ASD formulations were bioequivalent as they met 90% confidence of 90–11.1% for C_max_ and AUC. The CAD and ASD formulations exhibited C_max_ and AUC 1.7–1.8 and 1.5–1.8 folds of tablet formulations containing the drug’s crystalline phase. In conclusion, the stability, dissolution, and pharmacokinetic performance of SAIB-based CAD and HPMC-based ASD formulations were similar, and thus clinical performance would be similar.

## 1. Introduction

Most drugs fail to reach the market during development due to poor water solubility, even though they have desirable safety and efficacy profiles [1,2]. Several approaches were employed to enhance solubility and dissolution. These include chemical modification, particle size reduction, and formulation development [3,4]. Chemical modification of the drug results in significant changes in safety and efficacy profile that may result in elimination of potential lead compounds [3]. Particle size reduction improves dissolution rate but may not increase extent of crystalline solubility of drugs, even with an increase in surface-to-volume ratio [5,6]. Among the formulation development strategies, co-amorphous dispersion (CAD) and amorphous solid dispersion (ASD) are commonly used approaches for a poorly soluble drug where a crystalline drug is transformed into an amorphous form [7,8,9,10,11,12]. Dissolution of crystalline drugs includes disruption of the crystalline lattice, solvation/hydration, and breakdown of hydrogen bonds [13]. This approach exploits solubility advantage of an amorphous drug over a crystalline one due to lack of lattice order [14]. However, lack of lattice order increases free energy, making the amorphous form thermodynamically unstable. The amorphous drug reverts to stable crystalline form after high temperature and humidity exposure. The amorphous drug is stabilized to a certain extent against crystallization by adding a high melting/glass transition temperature polymer to form an ASD, which increases the glass transition temperature of the drug. The polymer also provides means of dosage form development and manufacturability [15]. CAD is formed when the polymer is replaced by a small molecule [7,8,11]. Small molecules stabilize the amorphous drugs through intermolecular interactions, e.g., hydrogen bonds, π–π, or even ionic etc. This technique is widely used for solubility, dissolution, and oral bioavailability enhancement of poorly water-soluble drugs [16,17]. The CAD and ASD can be formulated into tablet and or capsule dosage forms. FDA has approved many ASD since 2007 [18].

**Figure 1:**
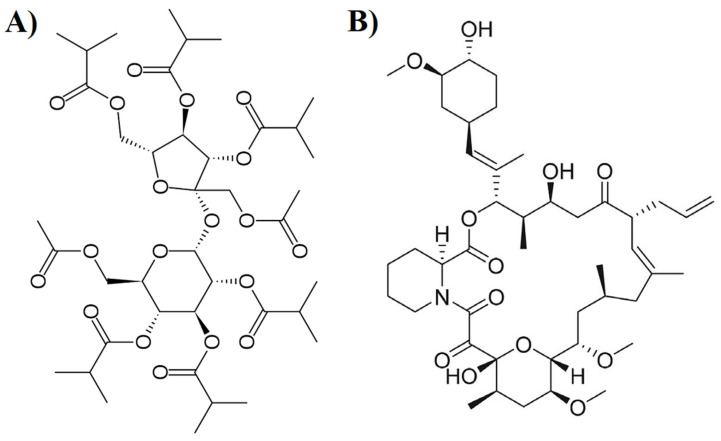
(**A**) Structure of SAIB, (**B**) Structure of Tacrolimus.

Tacrolimus (TAC) is a BCS class II drug, meaning solubility and dissolution is the rate-limiting step in its absorption [19]. The ASD of TAC has been reported to increase the oral bioavailability of the drug [9,20]. Immediate release ASD dosage form of TAC has been reported using hydroxypropyl cellulose [21], polyvinyl pyrrolidone, polyethylene glycol, hydroxypropyl methylcellulose (HPMC), [22], Eudragit^®^ [23], HPMC and sodium lauryl sulfate [24]. Similarly, extended-release ASD of TAC has also been reported using HPMC and ethyl cellulose [25,26]. Among all the reported polymers for ASD, HPMC was the most effective in maintaining supersaturation during in vitro and in vivo dissolution, thus, enhancing oral bioavailability [22]. Therefore, FDA approved immediate and extended-release ASD dosage forms of TAC. However, immediate and extended-release ASD formulations of TAC have been recalled due to failure to meet dissolution specification during stability testing [27,28]. This could be related to drug crystallization during stability if the formulation composition is not optimized, the manufacturing method is incorrect, and/or the packaging is defective, etc. Drug crystallization is a long-standing problem in ASD dosage forms [9,20]. This dictates to search for a new polymer or excipient that may inhibit or reduce the drug crystallization while maintaining supersaturation during dissolution and in vivo absorption. In this paper, an attempt was made to develop CAD of TAC using sucrose acetate isobutyrate (SAIB) as a new carrier and characterize for physicochemical, stability, and pharmacokinetic attributes and compared with HPMC-based ASD formulation. SAIB is a pale straw glassy solid at room temperature that liquefies at 60 °C. It is synthesized by controlled esterification of sucrose with acetic anhydride and isobutyric anhydride. The molecular formula and weight of SAIB are C_40_H_62_O_19_ and 846.9 g/mL, respectively (Figure 1A). It is insoluble in water and soluble in most organic solvents with a LogP ranging from 3.4 to 7. The calculated hydrogen donor and acceptor counts in the molecule is zero and 19, respectively [29]. The physicochemical properties of TAC are similar to SAIB (Figure 1B). The molecular weight and LogP values of the drug are 804.0 and 2.7, respectively. Unlike SAIB, TAC has eleven hydrogen acceptor and three hydrogen donor counts. Reported solubility of TAC in SAIB was 115 mg/gm [29]. It is possible that both molecules may form CAD by hydrogen and hydrophobic interactions based on structural and physicochemical attributes. SAIB has never been reported in the literature for CAD formulation development.

## 2. Materials and Methods

### 2.1. Materials

TAC monohydrate >98% and hydroxypropyl cellulose (HPC, MW 100,000) were purchased from Sigma-Aldrich (St Louis, MO, USA). Deuterated TAC-^13^C_3_D_2_ (>85%) and Beagle dog plasma were obtained from Toronto Research Chemicals, Ontario, Canada, and BioChemed Services, Winchester, VA, USA, respectively. BioSustane™ SAIB and microcrystalline cellulose (MCC, Avicel^®^ PH-101) were obtained from Eastman Chemical Company (Kingsport, TN, USA) and FMC Corporation (Princeton, NJ, USA), respectively. Hydroxypropyl methylcellulose (HPMC, 50 cps), magnesium stearate (MGS), croscarmellose sodium (CCS), lactose monohydrate, orthophosphoric acid (OPA), methanol, ammonium acetate, zinc sulfate (ZnSO_4_) and formic acid were purchased from Fisher Scientific (Asheville, NC, USA). Sodium lauryl sulfate (SLS) was purchased from VWR Chemicals, LLC (Fountain Parkway, OH, USA). All reagents were of analytical grade and used as received. In-house water (18 MΩcm, Millipore Milli-Q Gradient A-10 water purification system) was used in the study.

### 2.2. Preparation of CAD and ASD

The formulations were prepared by solvent evaporation method as per Table 1. Briefly, the drug and the SAIB or HPMC were dissolved/dispersed in ethanol by sonication, followed by the addition SLS with sonication, and MCC and CCS. Solvents were evaporated under the hood at room temperature with stirring to form a solid mass. The solid mass was crushed and passed through #60 sieve. The physical mixtures (PM) of CAD (F16) were prepared by adding the drug to the placebo formulation. Dried powder equivalent to 5 mg TAC either filled into hard gelatin capsules or mixed with MGS and compressed into tablets using a Mini Press-1 (Globe Pharma, New Brunswick, NJ, USA) 10-station tableting machine with 5-mm concave die and punches (Natoli Engineering Company, Saint Charles, MO, USA). A tablet formulation (F18) compositionally identical to F16 or F17 without SAIB or HPMC, and contained crystalline form of the drug was also prepared for pharmacokinetic study. Various solutions/dispersions of TAC and SAIB were also prepared by heating at 100–120 °C. All samples were stored in a desiccator until further analysis.

### 2.3. Fourier Transform Infrared Spectroscopy

The Fourier-transform infrared (FT-IR) spectra of the samples were collected by a modular NicoletTM iS™ 50 system (Thermo Fisher Scientific, Austin, TX, USA). The spectra were obtained in absorbance mode over a wavelength range of 400–4000 cm^−1^ with a data resolution of 8 cm^−1^ and 100 scans. A small amount of powder was placed on the diamond crystal and pressed with the attached arm to avoid any air entrapment in the sample. OMNIC software, version 9.0 (ThermoFisher Scientific), was used to capture and analyze the spectra.

### 2.4. X-ray Powder Diffractometry

X-ray powder diffraction (XRPD) of the samples was performed using Bruker D2 Phaser SSD 160 Diffractometer (Bruker AXS, Madison, WI) equipped with LYNXEYE scintillation detector and Cu Kα radiation (λ = 1.54184 Å) at a voltage of 30 KV and a current of 10 mA. Approximately 400 mg sample was evenly filled in a sample holder. The diffraction angle was set as 5 < 2θ < 15° at a rate of 2°/min and 1 s per step with an increment of 0.1778° and rotated at 15 rpm to collect average diffractograms. Data was evaluated using Diffrac. EVA Suite version V4.2.1 and further processed using File Exchange 5.0 (Bruker AXS, Madison, WI, USA).

### 2.5. Differential Scanning Calorimetry

The thermal behavior of formulation components, PM, CAD, and ASD, was assessed by differential scanning calorimetry (DSC) using Q2000 instruments (TA Instruments Co., New Castle, DE, USA). Approximately 5 mg sample was hermetically sealed in an aluminum pan. The samples were scanned over a temperature range of 10 to 250 °C at a rate of 10 °C/min to cover the melting point of the drug and excipients. Nitrogen gas was purged at a pressure of 20 psi and 50 mL/min flow rate to provide an inert atmosphere during the measurement.

### 2.6. Dissolution

Dissolution of the formulations was performed using USP dissolution apparatus 2 (Agilent 708-DS, Santa Clara, CA, USA) equipped with an autosampler (Agilent 850-DS Dissolution Sampling Station) in a 900 mL dissolution media (water containing 1 in 20,000 HPC, pH adjusted to 4.5) at 37 ± 0.5 °C and 50 rpm. The samples were withdrawn at 30, 60, 90, and 120 min, and the amount of drug dissolved was determined by the validated HPLC method. Dissolution samples were diluted with SLS solution (1%) in a ratio of 9:1 to ensure drug solubility and to prevent crystallization during analysis. The dissolution experiment was performed in triplicate.

### 2.7. Stability

Short-term stability of CAD formulation F16 (SAIB) and ASD formulation F17 (HPMC) was performed by packing in an HDPE bottle and storing at 25 °C/60% RH and 40 °C/75% RH for three months and one month, respectively. The samples were examined for physical and chemical changes by dissolution, FTIR, XRPD, and DSC.

### 2.8. Pharmacokinetics

This study was carried out to compare the pharmacokinetic profiles of HPMC-based ASD formulation (F17), SAIB-based CAD formulation (F16), and a tablet containing the crystalline form of the drug (F18). Four beagle dogs (2 males and 2 females, 10 ± 2 Kg) were used in this study. The study (IACUC 2019-0241) was approved by the Institutional Animal Care and Use Committee (IACUC) of Texas A&M University. The animals were given F16 (SAIB as a carrier), F17 (HPMC as a carrier), or crystalline drug tablets (F18) containing 5 mg TAC with 15 days washout period between the studies. The animals were fasted overnight (midnight to 7 a.m.) before administration of the dose and 2 h post-dosing. The animals had free access to water during fasting and food and water 2 h post-dosing. Lidocaine cream was applied to the catheterization site before catheterization/needle stick and applied at each puncture site. A bitter apple was applied to the catheter to prevent licking and chewing of the catheter, and E-collar was also used to prevent dogs from reaching the catheter. 3 mL of blood was collected at 0, 0.25, 0.5, 1, 2, 3, 4, 6, and 8 h through the cephalic or saphenous catheter. Blood samples at 12, 24, 36, 48, and 72 h were directly withdrawn from the cephalic or saphenous vein. Blood was immediately transferred to a heparinized tube and stored at −80 °C until analysis. The protein precipitation method was used to extract the drug from the sample. Whole blood samples (500 μL) were treated with 0.1 M ZnSO_4_ (50 μL) to break red blood cells, followed by addition of methanol (900 μL) and internal standard (IS) (100 μL TAC-^13^C_3_D_2_, 50 ng/mL). The samples were vortexed for 2 min and centrifuged at 13,300 rpm and 4 °C for 15 min. The supernatant was analyzed for TAC and IS by the UPLC-MS method.

### 2.9. High-Performance Liquid Chromatography

A reported HPLC method was modified and validated for dissolution and assay analysis of the formulations [30]. The HPLC consisted of Agilent 1260 series (Agilent Technologies, Wilmington, DE, USA) equipped with a quaternary pump, online degasser, column heater, autosampler, and UV/Vis detector. Separation of the analyte was achieved on a 4.6 × 150 mm, 3 µm Luna C18 (Phenomenex, Torrance, CA, USA) column and a C18, 4.6 × 2.5 mm (5 µm packing) Luna C18 guard column (Phenomenex, Torrance, CA). The mobile phase was ACN and 0.05 M phosphoric acid (65:35 *v*/*v*) flowing at 1.0 mL/min. The column and auto-sampler were maintained at 60 °C. A sample volume of 200 μL was injected into the system and detected at 210 nm. Two injections per sample were analyzed to demonstrate reproducibility of the method. Data was collected and analyzed using OpenLab software (Agilent Technologies, Wilmington, DE, USA).

### 2.10. Ultra-Performance Liquid Chromatography-Mass Spectroscopy

UPLC was performed on a Waters Acquity^®^ UPLC system (Waters Corporation, Milford, MA, USA) equipped with Agilent InfinityLab Poroshell 120 EC-C18 (4.6 × 50 mm, 2.7 μm) and maintained at 60 °C in the column oven. Separation of the analytes was performed using a mobile phase consisting of 5 mM ammonium acetate adjusted to pH 5 with formic acid and 0.1% formic acid (*v*/*v*) in methanol (5:95, *v*/*v*). The flow rate and run time were 0.5 mL/min, and 3 min, respectively. The retention times of TAC and TAC-^13^C_3_D_2_ were 1.405 and 1.402 min, respectively, and the peaks were well separated from the baseline. Mass spectrometry parameters were electrospray positive ionization (ESI+) mode with 0.8 KV capillary voltage and 15V collision energy. The TAC and TAC-^13^C_3_D_2_ molecular masses were detected at 804 and 806 Dalton by the QDa detector. The calibration range was 4-100 ng/mL. Concentrations of 4, 10, 50, and 100 ng/mL TAC were used in precision and accuracy assessment, and met the requirement of ±15% of nominal concentration. The method was validated per FDA bioanalytical method validation guidance [31].

## 3. Results and Discussion

### 3.1. Fourier Transformed Infrared Spectroscopy

**Figure 2:**
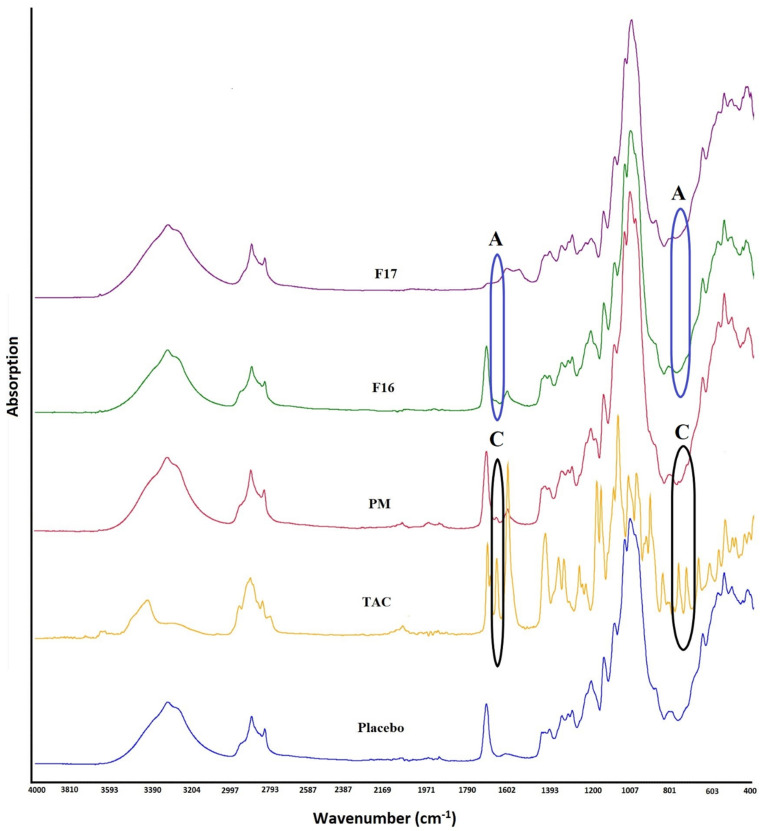
FTIR spectra of tacrolimus, physical mixture (PM), placebo, CAD, and ASD formulations (A: amorphous drug, C: Crystalline drug).

TAC showed absorption bands due to stretching vibrations of O-H at 3438 cm^−1^, C=O (ester and ketone) at 1738, 1723, and 1692 cm^−1^, C=O (keto-amide), and C=C at 1637 cm^−1^, C–O (ester) at 1172 cm^−1^, and C–O–C (ether) at 1087 cm^−1^. SAIB showed a strong absorption band at 1744 cm^−1^, which is related to its ester carbonyl. Major absorption bands of TAC appeared with reduced intensity in the PM due to dilution with the excipients. Furthermore, TAC absorption bands at 1738 and 1723 cm^−1^ were masked by SAIB at 1644 cm^−1^. In the case of CAD (F16), many characteristic absorption bands of TAC disappeared, broadened, or shifted to a new wavenumber. The absorption band of TAC at 783 and 1692 cm^−1^ disappeared, which may indicate the phase transformation of the drug. Similarly, F17, an ASD formulation based on HPMC, showed similar vibration bands (Figure 2). Phase transformation of the drug was further supported by XRPD and DSC data.

### 3.2. X-ray Powder Diffraction

**Figure 3:**
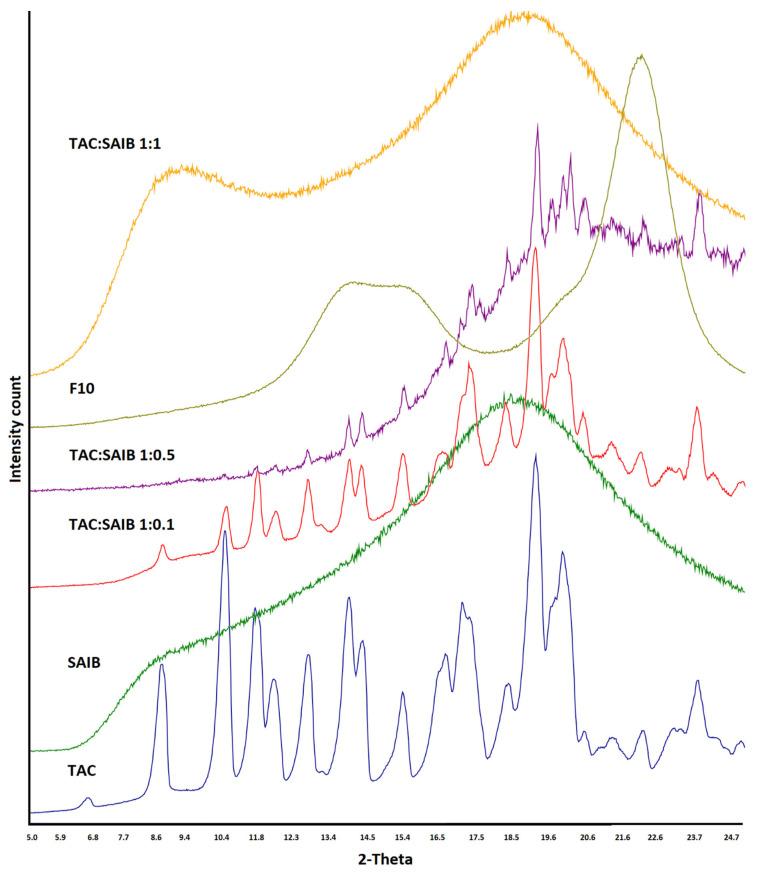
X-ray powder diffractogram of tacrolimus, SAIB, and various solutions/dispersions of drug and SAIB.

**Figure 4:**
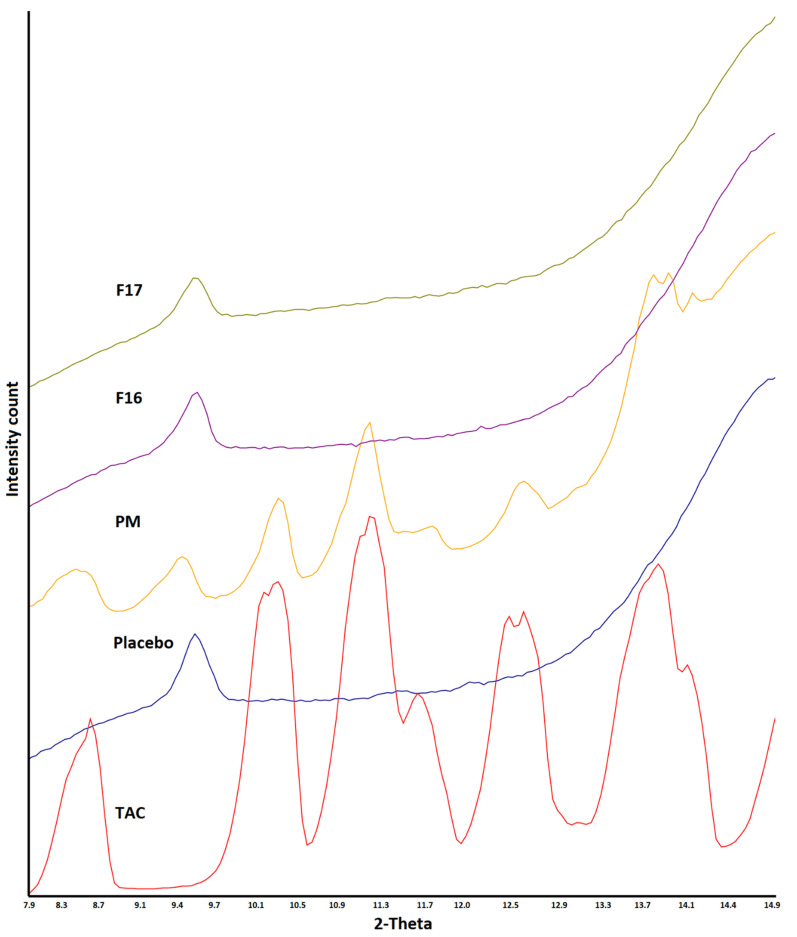
X-ray powder diffractogram, physical mixture, placebo, CAD, and ASD formulations.

TAC showed characteristics reflection peaks at 8.4, 10.2, 11.1, 11.6, 12.5, 13.6, 14.0, 15.2, 17.0, 18.0, 18.9, and 23.4° 2θ values. SAIB showed halo diffractograms, characteristics of amorphous or glassy materials. The degree of crystalline to amorphous phase ratio varied with TAC to SAIB. The crystalline phase decreased with SAIB content in the mixture. The crystalline phase completely disappeared when TAC: SAIB ratio was 1:1 or higher (Figure 3). Adding the excipients did not change the amorphous nature of the drug in the SAIB. XRPD was collected from 6 to 15° 2θ value as reflection peaks of the drug before 15° were not interfered with by the excipients (lactose, microcrystalline cellulose, etc.). Characteristics reflection peaks of the drug appeared with reduced peak height in the PM due to dilution with the excipients. In the case of F16 (SAIB) and F17 (HPMC) formulations, the drug peaks completely disappeared due to the formation of an amorphous phase (Figure 4).

### 3.3. Differential Scanning Calorimetry

**Figure 5:**
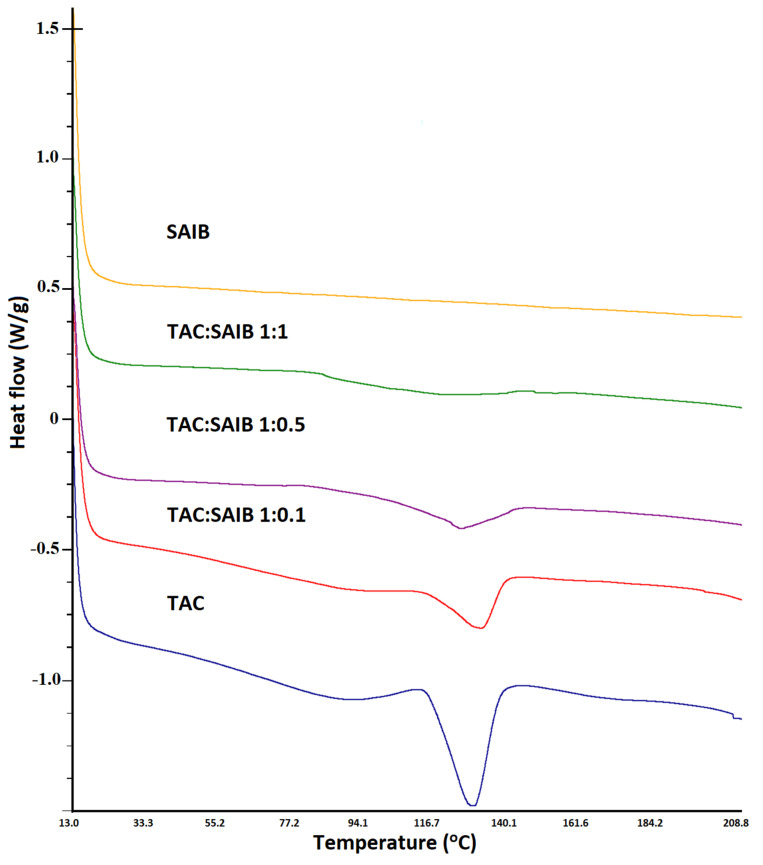
DSC thermograms of tacrolimus, SAIB, and various solutions/dispersions of drug and SAIB.

TAC showed a sharp melting endothermic peak at 131.5 °C that indicated the drug’s crystalline nature. The melting peak of the drug concurred with the literature-reported value [32]. SAIB showed no thermal event confirming its glassy nature. The addition of TAC to SAIB causes the transformation of the drug from crystalline to amorphous phase. However, the extent of phase transformation depends on the TAC to SAIB ratio. Height of the drug melting endothermic peak decreased with an increase in SAIB proportion in the formulation. This was due to drug solubilization in the glassy SAIB. The drug melting peak completely disappeared when TAC: SAIB ratio was 1:1 or higher (Figure 5).

**Figure 6:**
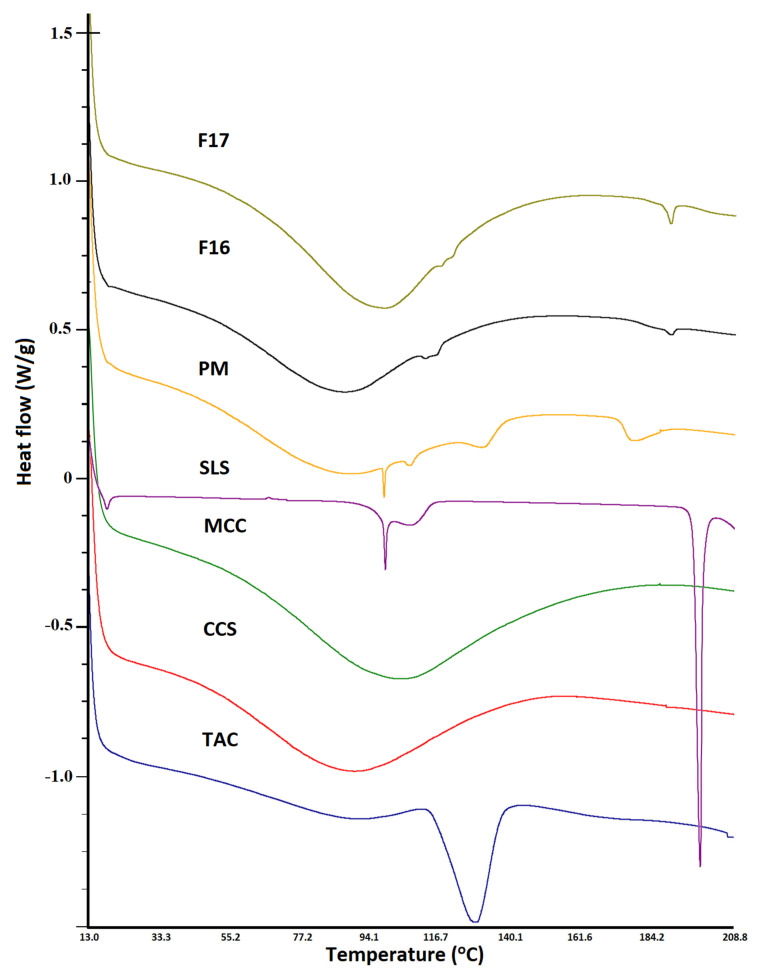
DSC thermograms of tacrolimus, physical mixture, placebo, CAD, and ASD formulations.

DSC thermogram of CCS and MCC exhibited shallow peaks in the region 60–130 °C, which could be due to physically adsorbed water. However, SLS showed multiple thermal events that may be due to its complex nature or the presence of numerous polymorphic forms. It is a mixture of sodium alkyl sulfate, mainly lauryl [33]. SLS showed thermal events at 103.9, 111.1, and 200.8 °C, which matched with the literature-reported values [34]. The PM showed additive thermograms with some differences from thermograms of individual components. The drug peak intensity was reduced due to dilution with excipients. Additionally, the drug melting peak was shifted to 134.2 °C due to excipients acting as impurities, thus causing a shift in melting point to a higher temperature. Additionally, the thermal event peak at 200.8 °C became broad and shifted to 180.8 °C. This was possibly due to the melting of the drug and partial dissolution that resulted in a broad peak at lower melting. The CAD and ASD formulations did not show an endothermic peak of the drug as observed in the PM, indicating conversion of the crystalline drug to the amorphous phase in formulations F16 and F17. However, a broad and shallow doublet appeared at 116–124 °C, which could be related to the glass transition temperature of the drug (Figure 6).

### 3.4. Dissolution

The dissolution specification of immediate release TAC capsule is 85% in 90 min in 900 mL dissolution media (water containing 1 in 20,000 HPC, pH adjusted to 4.5) as per USP [35]. This criterion was used to develop the CAD formulation of TAC. The drug dissolved in 2 h was 5.4 ± 0.9% from the pure TAC. Various ratios of TAC to SAIB and excipients were explored to increase rate and extent of the dissolution. F1 formulation contained a drug-to-SAIB ratio of 1:1. It showed almost no dissolution due to the hydrophobic nature of the drug and SAIB, and formed a blob in the dissolution medium. To disperse the blob and increase the surface area available for dissolution, LMH or MCC was added to the formulation in various ratios from 1:5 to 1:20 drug to LMH (F2 to F4) or MCC (F5 to F7). Dissolution was 1.9% in 2 h from the F3 formulation with TAC: SAIB: LMH ratio of 1:1:10. Increasing or decreasing the LMH in the formulations did not increase the dissolution. The dissolution was almost negligible when TAC: SAIB: LMH (F2) was 1:1:5. Increasing the LMH proportion in the formulation F4 from 1:10 to 1:20 did not significantly increase dissolution. The dissolution was 3.7 ± 1.4% in 2 h from F4 formulation, respectively.

**Figure 7:**
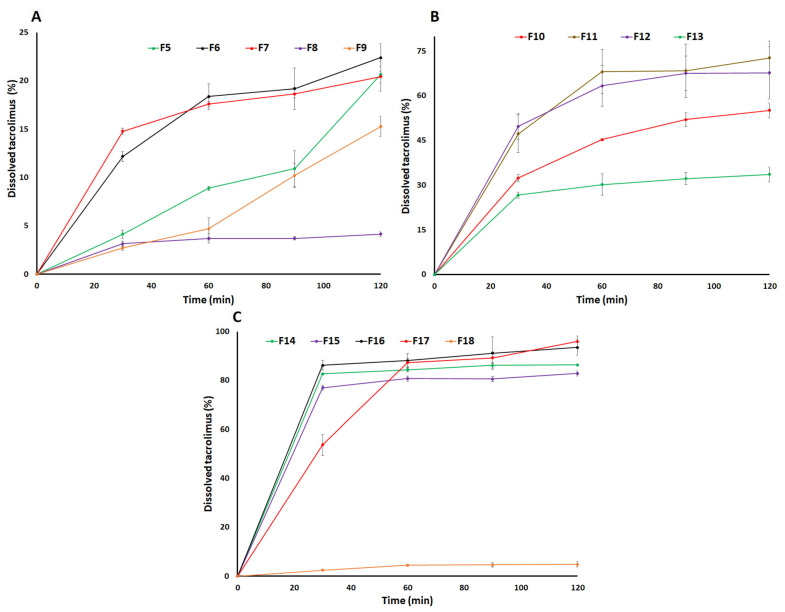
Dissolution profiles of (**A**) F5-F9, (**B**) F10-F13, and (**C**) F14-F18 formulations.

On the other hand, addition of MCC in the formulations caused a significant increase in dissolution compared to LMH-based formulations. Dissolution was 20.6 ± 0.9 and 22.4 ± 2.2% in 2 h from F5 and F6, respectively (Figure 7A). These formulations contained TAC: SAIB:MCC 1:1:5 and 1:1:10, respectively. The dissolution in MCC and LMH-based formulations can be explained by solubility, porosity/pore formation phenomenon, and adsorbed layer thickness. LMH must dissolve first to make pores for the dissolution medium to penetrate through the formulation matrix in order to dissolve the drug. This is not the case for MCC since it is porous, and a dissolution medium can easily penetrate through the matrix [36]. TAC-SAIB solution was physically adsorbed over the surface of LMH or MCC. The thickness of adsorbed TAC-SAIB layer would be thicker in LMH due to its non-porous nature. Thus, less surface area would be available compared to MCC-based formulations. Dissolution is proportional to the surface area as per the Noyes-Whitney equation [37]. Further, an increase in MCC in the formulation did not increase the extent of dissolution but increased the rate. For example, the drug dissolved was 20.4 ± 1.6% in 2 h in the F7 formulation (Figure 7A). A faster dissolution rate in the F7 formulation with an increase in MCC was due to the thinner adsorb layer of the TAC-SAIB solution. Thus, a higher surface area was available for the drug to dissolve.

To understand the effect of SAIB on the dissolution, TAC: SAIB proportion was decreased from 1:1 to 1:0.1 (F8) and 1:0.75 (F9) while keeping the proportion of TAC: MCC constant (1:10). Compared to F6, rate and extent of the dissolution in F8 and F9 decreased significantly. The dissolution was 4.1 ± 0.2 and 15.3 ± 1.0% in 2 h from F8 and F9, respectively (Figure 7A). A decrease in dissolution can be explained by crystallinity of the drug, which was supported by diffractograms and thermograms (Figure 3 and Figure 5). These formulations exhibited characteristic reflection peaks of the drug without MCC. Thus, a lower proportion of SAIB was insufficient to keep the drug in its molecular form. Dissolution was further increased by adding CCS outside the CAD formulation, which means CCS was added to the formulation after CAD manufacturing. Addition of CCS resulted in a significant increase in dissolution from 22.4 ± 2.2 (F6) to 50.2 ± 2.2% (F10) in 2 h (Figure 7B). Proportional ratio of TAC:SAIB:MCC:CCS was 1:1:10:2 in F10. An increase in dissolution in F10 can be explained by the fast dispersion of the capsule compact that resulted in a significant increase in rate and extent of the dissolution. The addition of CCS during the manufacturing step significantly impacted the dissolution. CCS was added during the CAD manufacturing step and, at the same time, increased the proportion of SAIB in the formulation. F11 formulation proportional composition was TAC:SAIB:MCC:CCS 1:1.5:10:2. The dissolution of TAC was increased from 50.2 ± 2.2 (F10) to 72.8 ± 5.6% (F11) in 2 h. Adding CCS during manufacturing step resulted in a thin coating of the formulation over the insoluble excipients, which increased the surface area available for dissolution. Further, an increase in SAIB proportion relative to the drug from 1:1.5 (F11) to 1:2 (F12) while keeping MCC and CCS constant did not increase dissolution. On the contrary, it decreased the dissolution, which can be explained by the hydrophobic nature of SAIB and the thick coating of the TAC-SAIB solution over the excipients and, thus, decreased available surface area for dissolution (Figure 7B). Surfactants were explored to increase rate and extent of the dissolution in formulation F11. SLS was added during the CAD manufacturing step in the ratio 1:1 ratio with respect to TAC in the formulation F13. The dissolution rate and extent were increased from 72.8 ± 5.6% (F11) to 81.3% in 2 h in F13 (Figure 7B). Surfactants are known to improve the dissolution of hydrophobic compounds by forming micelles structures [38]. Dissolution of 85% in 1.5 h can be achieved by increasing CCS proportion from 1 to 1.5 (F14) and 2 (F15) with respect to the drug. Increasing CCS resulted in 86.2 ± 1.4% dissolution in 1.5 h from the F14 formulation. However, increasing CCS to two proportions with respect to the drug in F15 did not result in a significant change in dissolution and did not achieve ≥85% dissolution in 1.5 h (Figure 7C). Formulation F14 was considered the optimized formulation that met the USP dissolution criterion [35]. Finally, F14 was converted from capsule to tablet dosage form to understand its impact on dissolution. The dissolution was increased from 86.2 ± 1.4 to 91.1 ± 6.6% in 1.5 h by changing the dosage forms (Figure 7C). This behavior can be explained by the tablet’s disintegration and the capsule’s bursting. The bursting time was about 5-10 min compared to less than 1 min disintegration time of the tablet. To compare with the HPMC formulation, the HPMC-based formulation (F17) was prepared by replacing the SAIB with HPMC and was compositionally identical to the F16 formulation. The dissolution was 89.2 ± 2.1% in 1.5 h from the HPMC-based formulation. The dissolution of tablet formulation with crystalline drug (F18) without SAIB or HPMC was 4.8 ± 1.1% in 2 h (Figure 7C).

### 3.5. Stability

**Figure 8:**
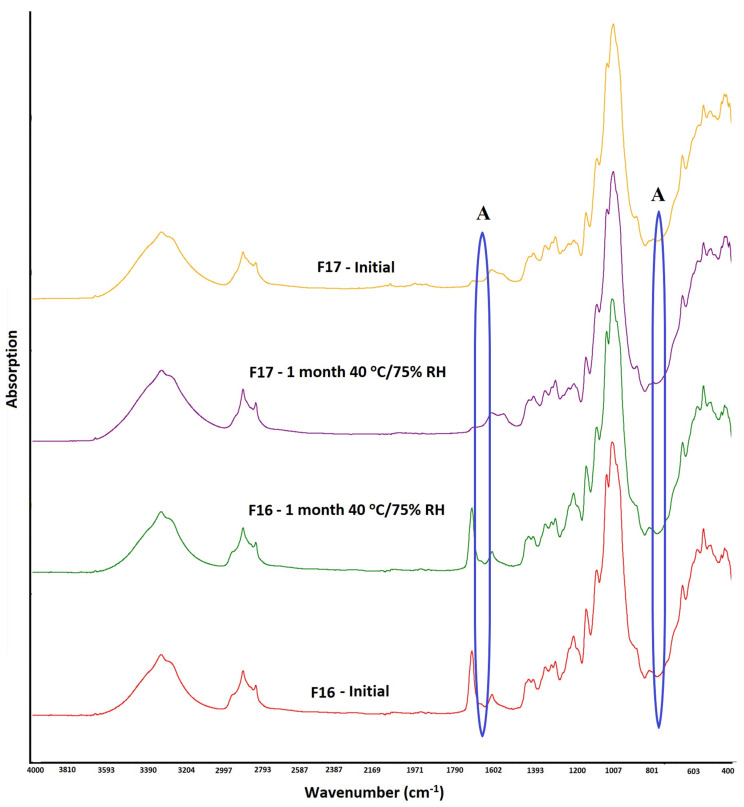
FTIR spectra of CAD and ASD formulations before and after storage at 25 °C/60% RH and 40 °C/75% RH conditions (A: amorphous drug).

The stability conditions exposed samples showed no significant changes in the physical and chemical attributes of the CAD and ASD formulations. FTIR spectrum indicated insignificant changes in the F16 formulation on exposure to high humidity and temperature conditions, which suggested no chemical interactions and maintenance of the amorphous phase of the drug during storage. This was supported by the nonappearance of the absorption bands of TAC at 783 and 1692 cm^−1^. Similar findings were observed in the F17 formulation, and the spectrum was identical to before exposed sample (Figure 8).

**Figure 9:**
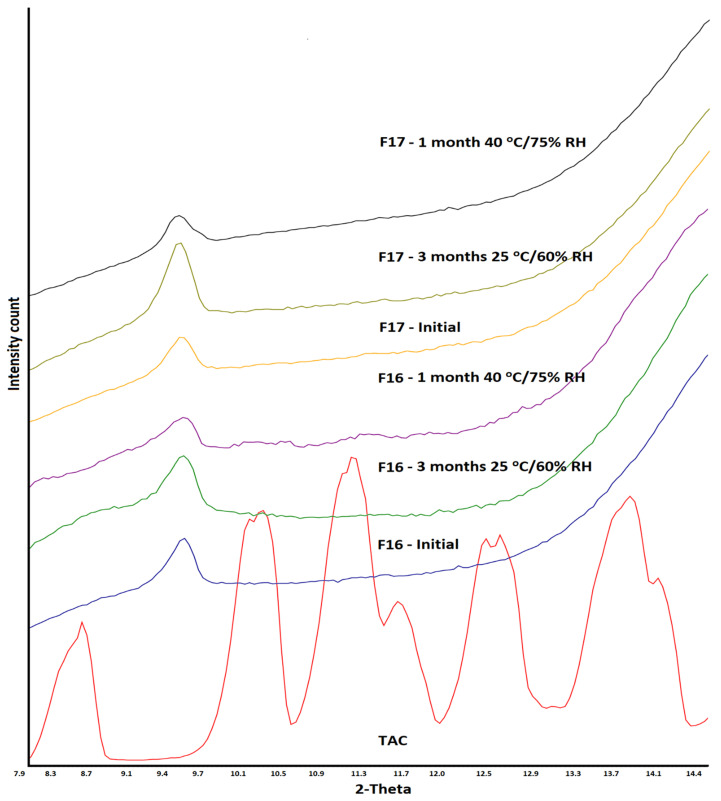
X-ray powder diffractogram of CAD and ASD formulations before and after storage at 25 °C/60% RH and 40 °C/75% RH conditions.

**Figure 10:**
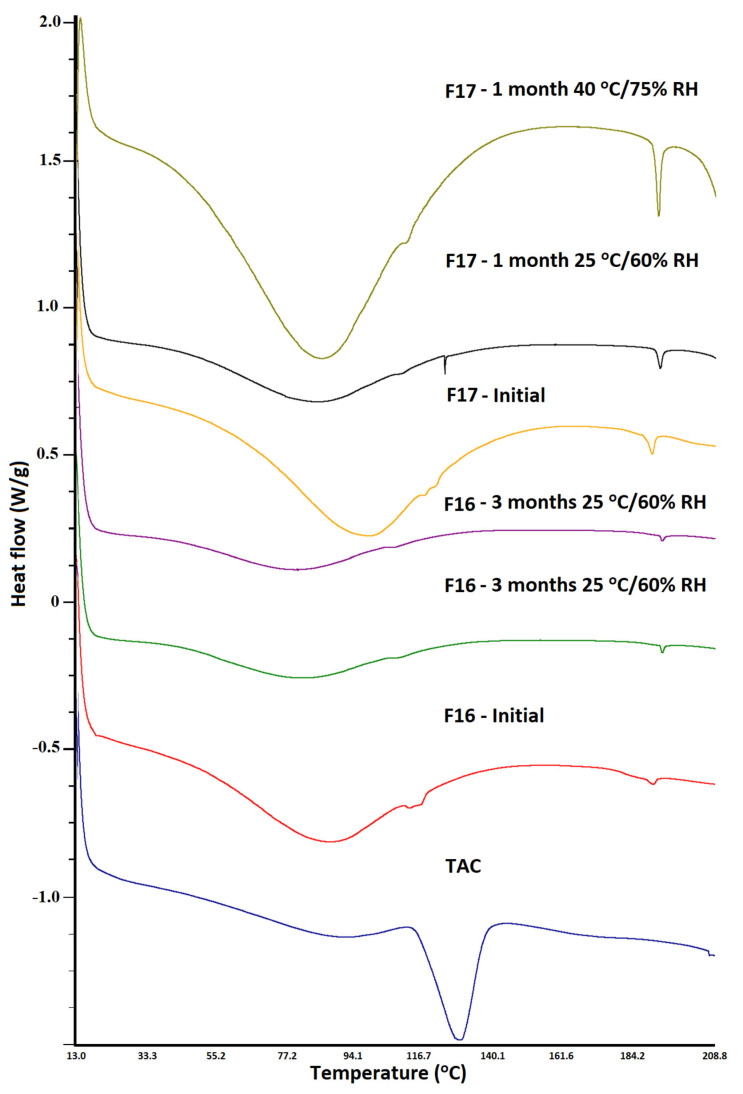
DSC thermograms of CAD and ASD formulations before and after storage at 25 °C/60% RH and 40 °C/75% RH conditions.

This was further supported by DSC and XRPD data. The diffractogram of F16 and F17 formulations did not show appearance of characteristics reflection peak of the drug at 8.4, 10.2, 11.1, 11.6, 12.5, and 13.6° that could have indicated amorphous to crystalline reversion (Figure 9). Similarly, the melting peak of the drug in the region 130–135 °C did not appear in thermograms of the exposed F16 and F17 formulations (Figure 10). The data of orthogonal techniques indicated no transformation of the amorphous drug to its crystalline form on exposure to high humidity and temperature condition.

**Figure 11:**
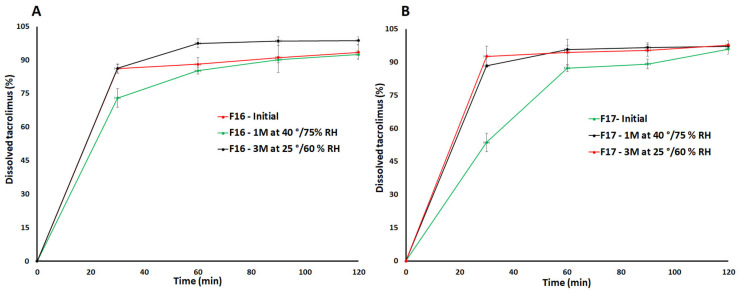
Dissolution profiles of (**A**) F16 and (**B**) F17 formulations before and after storage at 25 °C/60% RH and 40 °C/75% RH conditions.

No significant change in dissolution was expected as no reversion of the drug phase was observed. Both formulations (F16 and F17) met the USP limit of 85% in 90 min after exposure to stability conditions (Figure 11). The dissolution changed from 91.1 ± 6.6% to 90.1 ± 1.8 and 98.5 ± 1.9% after exposure of F16 to 25 °C/60% and 40 °C/75% RH, respectively. Similarly, the dissolution changed from 89.2 ± 2.2% to 96.6 ± 0.2 and 95.4 ± 2.7% after exposure of F17 to stability conditions. An increase in the dissolution rate was observed at 30 min, possibly due to decreased disintegration time after exposure.

### 3.6. Pharmacokinetics

**Figure 12:**
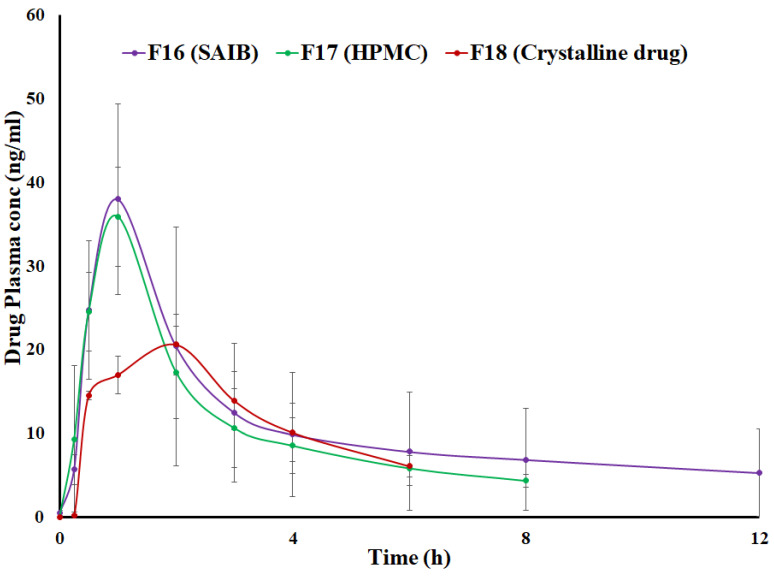
Comparative pharmacokinetic profiles of F16 (SAIB), F17 (HPMC), and F18 formulations.

Oral absorption of TAC is incomplete and variable. The absolute bioavailability of TAC is 17 ± 10% in adult kidney transplant patients, 22 ± 6% in adult liver transplant patients, 23 ± 9% in adult heart transplant patients, and 18 ± 5% in healthy volunteers [39]. Comparative bioequivalence was performed among F16 (SAIB-based CAD), F17 (HPMC-based ASD), and Control formulation (F18 formulation contained crystalline drug without SAIB or HPMC). The pharmacokinetic profiles of F16 and F17 formulations were similar and almost superimposable but different from F18 (Figure 12). The drug was detectable up to 72 h post-dosing but below the quantification limit of 4 ng/mL after 12, 8, and 6 h from F16, F17, and F18, respectively.

The pharmacokinetic parameters of F16 and F17 were also very similar. Maximum plasma concentration (C_max_) and AUC_0–∞_ (area under the plasma concentration curve) of F16 and F17 were 38.0 ± 11.4 and 35.9 ± 5.9 ng/mL, and 270.3 ± 222.2 and 224.7 ± 55.3 ng/mL.h, respectively. The time to achieve (T_max_) C_max_ was one hour for both formulations. No statistical differences were observed in the pharmacokinetic parameters of both formulations (*p* < 0.05). On the other hand, the pharmacokinetic profile of F18 was completely different from CAD and ASD formulations. Time to achieve C_max_ was longer in PM compared to CAD and ASD formulations, and was reached in 2 h. Furthermore, the AUC of F18 was lower than F16 and F17 formulations which also concurred with dissolution data. Longer T_max_ and lower C_max_ and AUC_0–∞_ value in F18 were due to crystalline drug that accounted for lower in vitro and in vivo dissolution compared to CAD and ASD formulations. CAD and ASD formulation can achieve supersaturation due to the amorphous nature of the drug [5,40,41]. T_max_, C_max_, and AUC_0–∞_ of F18 were two h, 20.6 ± 0.6 ng/mL, and 142.9 ± 10.8 ng/mL.h, respectively. The formulations (F16 and F17) exhibited C_max_ and AUC 1.7–1.8 and 1.5–1.8 folds of the F18 formulation, respectively. Higher values of pharmacokinetic parameters in CAD and ASD formulations were due to faster and higher dissolution. Dissolution is the rate-determining step in the absorption and bioavailability of BCS class II drugs such as TAC [9,42]. Furthermore, statistically significant differences (*p* < 0.05) were observed between the pharmacokinetic parameters of CAD and ASD (F16 and F17) and F18 formulations. Therefore, more drug is expected to be bioavailable from CAD and ASD formulations compared to F18.

Furthermore, FDA standards were used to determine bioequivalence among F16, F17, and F18 formulations. The two formulations were considered bioequivalent when the confidence interval (CI) at the alpha level of 90% is 80–125% of the geometric mean of the log-transformed ratio of test/reference or reference/test of C_max_ and AUC for regular drug [43]. However, the CI criterion is tightened for narrow therapeutic index (NTI) drugs to ensure clinical similarity when formulations are switched. FDA-recommended CI criterion is 90–11.1% for NTI [44,45]. The ratio of C_max_ of F16/F17 and F17/F18 ranged from 99.7 to 100.3%. Similarly, the AUC of F16/F17 and F17/F18 ranged from 98.0 to 102.0%. Therefore, the F16 and F17 formulations can be considered bioequivalent as they met the CI criterion for both pharmacokinetic parameters. Thus, the clinical performance of F16 would be similar to F17, and these formulations may be interchangeable.

Bioequivalence of the F16 or F17 formulation was also compared with the F18 formulation. The ratio of C_max_ and AUC of F16 /F18 and F18/F16 ranged from 84.3 to 118.7 and 90.2 to 110.9%, respectively. Both C_max_ and AUC criteria should be met to be considered bioequivalent. Thus, F16 and F18 were not bioequivalent. F17 and F18 were not bioequivalent and interchangeable as they did not meet the CI criteria of pharmacokinetic parameters. The ratio of C_max_ and AUC of F17/F18 and F18/F17 ranged from 84.6 to 118.3 and 90.0 to 108.7%, respectively.

## 4. Conclusions

CAD and ASD formulations offer a tremendous opportunity to convert the poorly insoluble drug into a soluble, dissolvable, and absorbable molecule, provided it maintains its amorphous phase at various stability conditions. The prepared SAIB-based CAD formulation showed that the drug was in the amorphous phase as indicated by XRPD and DSC data. The dissolution can be modulated by SAIB proportion and other excipients to meet regulatory or pharmacopeial requirements. Short-term stability at room temperature and accelerated conditions indicated that the drug’s amorphous phase was maintained and met dissolution specifications. The dissolution and stability of the SAIB-based CAD formulation were similar to the HPMC-based ASD formulation. Therefore, the pharmacokinetic and clinical performance of SAIB-based CAD would be similar to HPMC-based ASD formulations. However, long-term stability and human study data must correlate with the current findings.

## Figures and Tables

**Table 1:** Composition of tacrolimus CAD and ASD formulations.

Formulation	Tacrolimus (mg)	SAIB (mg)	SLS (mg)	MCC (mg)	LMH (mg)	HPMC (mg)	CCS (mg)	Dosage Form
F1	5	5	0	0	0	0	0	Capsule
F2	5	5	0	0	25	0	0	Capsule
F3	5	5	0	0	50	0	0	Capsule
F4	5	5	0	0	100	0	0	Capsule
F5	5	5	0	25	0	0	0	Capsule
F6	5	5	0	50	0	0	0	Capsule
F7	5	5	0	100	0	0	0	Capsule
F8	5	0.5	0	50	0	0	0	Capsule
F9	5	3.75	0	50	0	0	0	Capsule
F10	5	5	0	50	0	0	10	Capsule
F11	5	7.5	0	50	0	0	10	Capsule
F12	5	10	0	50	0	0	10	Capsule
F13	5	7.5	5	50	0	0	10	Capsule
F14	5	7.5	5	50	0	0	15	Capsule
F15	5	7.5	5	50	0	0	20	Capsule
F16	5	7.5	5	50	0	0	15	Tablet
F17	5	0	5	50	0	7.5	15	Tablet
F18	5	0	5	50	0	0	15	Tablet
